# Hemoglobin and Hematocrit Levels in the Prediction of Complicated Crohn's Disease Behavior – A Cohort Study

**DOI:** 10.1371/journal.pone.0104706

**Published:** 2014-08-12

**Authors:** Florian Rieder, Gisela Paul, Elisabeth Schnoy, Stephan Schleder, Alexandra Wolf, Florian Kamm, Andrea Dirmeier, Ulrike Strauch, Florian Obermeier, Rocio Lopez, Jean-Paul Achkar, Gerhard Rogler, Frank Klebl

**Affiliations:** 1 Department of Internal Medicine I, University of Regensburg, Regensburg, Germany; 2 Department of Quantitative Health Sciences, Cleveland Clinic Foundation, Cleveland, Ohio, United States of America; 3 Division of Gastroenterology and Hepatology, University Hospital, Zurich, Switzerland; 4 Department of Pathobiology, Lerner Research Institute, Cleveland Clinic Foundation, Cleveland, Ohio, United States of America; 5 Department of Gastroenterology, Hepatology & Nutrition, Cleveland Clinic, Cleveland, Ohio, United States of America; University Hospital Llandough, United Kingdom

## Abstract

**Background:**

Markers that predict the occurrence of a complicated disease behavior in patients with Crohn's disease (CD) can permit a more aggressive therapeutic regimen for patients at risk. The aim of this cohort study was to test the blood levels of hemoglobin (Hgb) and hematocrit (Hct) for the prediction of complicated CD behavior and CD related surgery in an adult patient population.

**Methods:**

Blood samples of 62 CD patients of the German Inflammatory Bowel Disease-network “Kompetenznetz CED” were tested for the levels of Hgb and Hct prior to the occurrence of complicated disease behavior or CD related surgery. The relation of these markers and clinical events was studied using Kaplan-Meier survival analysis and adjusted COX-proportional hazard regression models.

**Results:**

The median follow-up time was 55.8 months. Of the 62 CD patients without any previous complication or surgery 34% developed a complication and/or underwent CD related surgery. Low Hgb or Hct levels were independent predictors of a shorter time to occurrence of the first complication or CD related surgery. This was true for early as well as late occurring complications. Stable low Hgb or Hct during serial follow-up measurements had a higher frequency of complications compared to patients with a stable normal Hgb or Hct, respectively.

**Conclusions:**

Determination of Hgb or Hct in complication and surgery naïve CD patients might serve as an additional tool for the prediction of complicated disease behavior.

## Introduction

Crohn's disease (CD) is frequently characterized by the occurrence of a complicated disease behavior, defined as fistulae or stenoses, and the need for CD related surgery. Up to two thirds of the CD patients develop strictures or fistulizing complications within ten years of diagnosis [1], [2]. A large proportion of CD patients have to undergo CD related surgery at least once during their lifetime and postoperative recurrence often occurs followed by multiple surgical interventions [3]. During recent decades better control of CD associated intestinal inflammation could be achieved, due to the emergence of stronger and more selective immunosuppressive therapies and immunomodulators. Therefore clinicians desire tools to monitor for patients that have a higher susceptibility for the development of complicated disease behavior or CD related surgery as this can impact therapeutic management.

Serological markers directed against microbial peptides and linked to CD, such as anti-*Saccharomyces cervisiae* (ASCA) and others have been extensively investigated for disease stratification and association [4]–[6]. Limited information is available on the *predictive* abilities of serum markers, mainly from pediatric cohorts, indicating an increased hazard for complicated disease behavior and CD related surgery with an increasing immune response to microbial components [7]–[9]. The still limited accuracy of the above-mentioned serologic markers as well as the high costs of their determination restricts their use in clinical practice. While red cell parameters have been linked to IBD activity [10]–[12], comparably little attention has been given to hemoglobin (Hgb) or hematocrit (Hct) for disease course prediction. An exploration of these markers is warranted as one could speculate that more severe disease or tissue damage is likely to be linked to anemia.

The specific aim of this cohort study was to evaluate the levels of Hgb and Hct early in the disease course as well as during follow-up visits to an IBD center as predictive markers for complicated CD behavior and CD related surgery in a well-defined German cohort.

## Materials and Methods

### Patient population

We performed a cohort study among adult CD patients. The diagnosis of CD was made based on clinical, radiographic, endoscopic and histopathologic criteria [13]. All CD in- and outpatients seen at our clinic between 2000 and 2006 were asked at study entry to donate blood for our repository. Exclusion criteria for this study were A) the presence of complicated disease, defined as fistula or stricture, or intestinal surgery at the time of first sample procurement B) a follow-up of less than three years for patients with a pure inflammatory disease course and C) missing values for blood Hgb or Hct. The patients with a pure inflammatory disease course and a follow-up period of less than three years were excluded to ensure a long enough follow-up time for the development of a possible complication. The complete repository consists of 363 individual CD patients. Blood samples before complication or surgery with a minimum follow-up of three years were available in 76 patients, of which 63 had values for Hgb or Hct recorded in our database. Blood was procured at multiple time points from 34 out of the included 62 CD patients during their disease course at arbitrary visits to our IBD unit or hospital allowing a longitudinal analysis.

Full clinical data including age at diagnosis, BMI, gender, date of sample procurement, date and type of complications and surgery, disease location and disease status were obtained for the time point of first sample procurement and later updated at each arbitrary visit thereafter separately by the treating physician of the IBD unit. Once collected, data were transferred and stored in a secure coded anonymized database for analysis. Disease activity was determined by the treating IBD physician and patients were grouped in active and non-active disease, based on clinical criteria as represented in the Crohn's disease activity index (CDAI). A CDAI point value of >150 was considered active disease and a point value ≤150 was considered inactive disease. We did not secure an exact CDAI score in our database. The disease activity evaluation was performed by four experienced IBD physicians for the majority of the patients. Iron supplementation therapy at time of initial sample procurement or during follow-up as well as number of blood transfusions during the study period were recorded. Anemia treatment was at the discretion of the IBD physician. We additionally procured information on the type of anemia present, separating the patients into iron-deficiency anemia, anemia of chronic disease, B12 or folate deficiency and renal anemia. At time of study analysis all patient charts and the database were reviewed and updated for the clinical data points. Follow-up for a particular patient was terminated if there was no further record available.

### Ethics statement

Signed informed consent was obtained from all participants. The ethics committee of the University of Regensburg approved the study.

### Blood analysis

All blood and sera values were measured according to standard laboratory procedures of the Department of Clinical Chemistry of the University of Regensburg. Standard laboratory values were defined as: Hgb abnormal for <13.3 g/dl for males and <11.2 g/dl for females; Hct abnormal for <40.1% for males and <34.1% for females; Albumin abnormal for <34 g/l. Anti *Saccharomyces cervisiae* IgG antibody was measured in the serum as previously described [6].

### Phenotypical characteristics of IBD patients

The treating physician assessed the CD patients for disease phenotype at arbitrary visits during the disease course. Patient demographics at study entry (time of first sample procurement/baseline) are given in **table 1**. For the purpose of this study complicated disease behavior in CD patients was defined as the diagnosis of fistulae or stenoses during follow up. We did not distinguish internal penetrating from perianal fistulizing disease. Furthermore, we examined the need for CD related abdominal surgery during the follow-up period, which included abdominal surgery as well as perianal surgery.

**Table 1:** Cohort Characteristics.

Factor	All	Low Hct	Normal Hct		Low Hgb	Normal Hgb	
	(N = 62)	(N = 17)	(N = 45)	p-value	(N = 30)	(N = 32)	p-value
Female	33 (53.2)	11 (64.7)	22 (48.9)	0.27	22 (73.3)	11 (34.4)	***0.002***
Mean age at study (SD)	32.5±11.9	33.0±14.7	32.4±10.8	0.85	31.6±12.3	33.4±11.6	0.54
Mean BMI (kg/m2) (SD)	23.0±3.6	21.6±3.8	23.5±3.5	0.064	21.8±3.4	24.2±3.5	***0.009***
Mean age at diagnosis (SD)	29.6±11	29.8±14.1	29.5±9.7	0.93	29.0±11.6	30.1±10.5	0.71
Median disease duration (months) (P25, P75)	16.5 [1.8,50.4]	7.8 [1.02,46.3]	21.7 [1.8,50.4]	0.43	7.2 [1.02,38.0]	27.8 [3.8,66.8]	0.062
Ileum involvement	51 (82.3)	13 (76.5)	38 (84.4)	0.46	25 (83.3)	26 (81.3)	0.83
Clinically active disease	18 (29)	7 (41.2)	11 (24.4)	0.20	8 (26.7)	10 (31.3)	0.69
CRP>0.5 mg/dl	32 (51.6)	12 (70.6)	20 (44.4)	0.066	19 (63.3)	13 (40.6)	0.074
Medications at time of first sample procurement							
5-ASA	33 (53.2)	4 (23.5)	29 (64.4)	***0.004***	13 (43.3)	20 (62.5)	0.13
Sulfasalazine	2 (3.2)	0 (0.0)	2 (4.4)	0.99	0 (0.0)	2 (6.3)	0.49
Corticosteroids	30 (48.3)	10 (58.8)	20 (44.4)	0.67	25 (50.0)	15 (46.8)	0.53
Azathioprine	7 (11.3)	0 (0.0)	7 (15.6)	0.084	2 (6.7)	5 (15.6)	0.43
Methotrexate	9 (14.5)	2 (11.8)	7 (15.6)	0.71	3 (10.0)	6 (18.8)	0.48
Medications at any time during follow-up							
5-ASA	39 (62.9)	8 (47.1)	31 (68.9)	0.11	17 (56.7)	22 (68.8)	0.32
Sulfasalazine	7 (11.3)	1 (5.9)	6 (13.3)	0.41	1 (3.3)	6 (18.8)	0.10
Corticosteroids	41 (66.1)	13 (76.4)	28 (62.2)	0.69	18 (60.0)	23 (71.9)	0.88
Azathioprine	13 (21)	1 (5.9)	12 (26.7)	0.073	3 (10.0)	10 (31.3)	***0.040***
Methotrexate	9 (14.5)	2 (11.8)	7 (15.6)	0.71	3 (10.0)	6 (18.8)	0.48
Extraintestinal manifestations at any time during follow-up							
Any	21 (33.9)	7 (41.2)	14 (31.1)	0.46	9 (30.0)	12 (37.5)	0.53
Joint	17 (27.4)	5 (29.4)	12 (26.7)	0.99	7 (23.3)	10 (31.3)	0.49
Skin	2 (3.2)	2 (11.8)	0 (0.0)	0.072	2 (6.7)	0 (0.0)	0.23
Eye	1 (1.6)	1 (5.9)	0 (0.0)	0.28	1 (3.3)	0 (0.0)	0.49
PSC	1 (1.6)	0 (0.0)	1 (2.2)	0.99	0 (0.0)	1 (3.1)	0.99
Vienna classification				***0.039***			***0.039F***
Inflammatory	44 (71.0)	8 (47.1)	36 (80.0)		17 (56.7)	27 (84.4)	
Stricturizing	8 (12.9)	4 (23.5)	4 (8.9)		5 (16.7)	2 (6.3)	
Fistulizing	10 (16.1)	5 (29.4)	5 (11.1)		8 (26.7)	3 (9.4)	
Montreal classification				***0.024F***			0.056F
B1	44 (71.0)	8 (47.1)	36 (80.0)		17 (56.7)	27 (84.4)	
B1p	6 (9.7)	2 (11.8)	4 (8.9)		4 (13.3)	2 (6.3)	
B2	8 (12.9)	4 (23.5)	4 (8.9)		5 (16.7)	3 (9.4)	
B3	4 (6.5)	3 (17.7)	1 (2.2)		4 (13.3)	0 (0.0)	
Surgery	13 (21.0)	7 (41.2)	6 (13.3)	***0.016***	10 (33.3)	3 (9.4)	***0.021***
Complication and/or surgery	20 (32.2)	10 (58.8)	10 (22.2)	***0.006***	14 (46.7)	6 (18.8)	***0.019***
Median time from sample procurement to 1st event or last FU (P25, P75)	55.8 [31.9,70.2]	31.9 [7.8,55.9]	60.6 [49.0,73.6]	***0.003***	46.3 [13.1,56.7]	62.4 [51.7,78.8]	***<0.001***

Values are depicted as n (%), unless otherwise stated.

BMI, body mass index; CD: Crohn's disease; Hct:hematocrit; Hgb: hemoglobin.

P25, P75: 25th and 75th percentiles; SD: standard deviation; FU: follow-up; pos.: positive; neg.: negative.

Low Hct: <40% for males and <37% for females.

Low Hb: <14.2 g/dL for males and <13.3 g/dL for females.

p-value correspond to t-tests for age and BMI, Wilcoxon rank sum test for disease duration and follow-up time, Fisher's Exact test if denoted by F and Pearson's chi-square otherwise.

### Statistical analysis

Descriptive statistics were computed for all variables. In order to assess differences between subjects with complications or CD related surgery and those without, Student's t-tests were used for age and BMI, Wilcoxon rank sum tests were used for disease duration, and Pearson's chi-square test was applied for all categorical variables. A time-to-event analysis was performed to assess whether marker status was associated with occurrence of 1^st^ complication or surgery (defined as an event). Time-to-event analysis was performed to assess factors associated with occurrence of first complications and/or surgery (defined as an event). Time of follow-up was defined as months between time of marker determination and occurrence of 1^st^ event or last record of patient history if no events were observed. Kaplan-Meier plots were generated and log-rank tests were used to assess differences in event-free rates between groups. In addition, univariable and multivariable Cox proportional hazards models were used and the hazard ratios (HR) and 95% confidence intervals for each marker were estimated to assess association between low hemoglobin levels and occurrence of any event while adjusting for potential confounders (ileum involvement, active disease, disease duration, age, early disease onset, CRP and ASCA) one at a time; the same was done for low hematocrit levels. SAS version 9.2 software (The SAS Institute, Cary, NC) and R version 2.9.1 software (The R Institute for Statistical Computing, Vienna, Austria) were used for all analyses. A p<0.05 was considered statistically significant.

## Results

### Clinical phenotypes of the population

A total of 63 patients were included in this study. One patient developed a hematologic malignancy (non-Hodgkin lymphoma (NHL)) after end of follow-up. To ensure no subclinical influence of the NHL on the CD course this patient was excluded from the study. The median time from diagnosis to study entry (first sample procurement/baseline) was 16.5 months (25^th^ percentile (P25) 1.8 months; 75^th^ percentile (P75) 50.4 months) with 38.7% of the samples procured within 6 months and 50% within one year of diagnosis of CD. Among the patients studied a total of 34% experienced a complication or CD related surgery during a median of 55.8 months of follow-up: 11.1% progressed to complication only, 17.5% to complication and surgery combined, and 3.2% had to undergo CD related surgery only. In the patients with complications 6 perianal fistulae, 4 internal penetrating fistulae and 13 stenoses occurred. The reasons for surgery were perianal surgery in 5, abdominal surgery in 5 and both types of surgery in 4 patients. The reason for surgery in the 2 patients without a previous complication was abscess surgery without a fistula in the pathology report in the first subject and ileocecal resection without a stricture or fistula in the pathology report in the second subject. At the time of sample procurement 64.5% of the CD patients were taking immunosuppressive medication, defined as any one out of corticosteroids (including budesonide), azathioprine or methotrexate. While use of anti-tumor necrosis factor-α agents was not an exclusion criterion, no patient in this cohort received infliximab or other anti-tumor necrosis factor-α agents at time of sample procurement or during follow-up. In the CD patients with anemia based on the laboratory standard values at our University Center 44.4% has iron deficiency anemia, 22.2% anemia of chronic disease, and 11.1% each had renal disease related anemia, iron deficieny anemia and B12 deficiency combined or an unknown cause. Serum albumin measurement was not performed in 6 out of 62 patients and was normal in all other 56 patients at time of first sample procurement. 5 out of 63 patients were on iron supplementation therapy at time of initial sample procurement and an additional 14 patients received iron supplementation at any time during follow-up. One patient had a trauma during follow-up and was resuscitated with 4 units of PRBC.

As there are no established criteria for using Hgb and Hct for disease prediction in CD, we next assessed their optimal cut-off values for prediction of complicated CD behavior or CD related surgery. We the used %findcut SAS macros developed at the Mayo Clinic [14] to determine the optimal cut-off for Hgb and Hct separately for male and female study participants. Based on log-rank statistics for time-to-event analysis a cut-off value of Hgb<14.2 g/dl for males and <13.3 g/dl for females and of Hct <40% for males and <37% for females was found to be optimal. According to these cut-off values neither a low Hgb nor a low Hct were associated with age at diagnosis, age at sample procurement, disease duration, ileal involvement or the intake of immunosuppressive medication. The median time from sample procurement to the first event was shorter in patients with low Hgb (31.9 months; P25 7.8, P75 55.9 months) compared to normal Hgb (60.6 months; P25 49, P75 73.6 months; p = 0.003) and shorter in patients with low Hct compared (46.3 months; P25 13.1, P75 56.7 months) to normal Hct (62.5 months; P25 51.7, P75 78.8 months; p<0.001). A high proportion of patients with a normal Hct were on 5-ASA compared to low Hct (62.2 versus 23.5%; p = 0.004). In addition a normal Hct or Hgb were linked to a higher proportion of patients with purely inflammatory disease behavior or Vienna B1 (**table 1**). Considering all included samples, the levels of Hgb and Hct only weakly correlated with CRP levels (rho = −0.30 and −0.28, respectively). No association for Hgb/Hct could be detected with (1) use of corticosteroids (yes vs. no: 13.5±1.6 vs. 14.0±1.4 g/dl; p = 0.21 and 39.5±4.1 vs. 40.5±3.8%; p = 0.37, respectively), (2) use of other immunosuppressants (yes vs. no: 13.6±1.5 vs. 14.0±1.5 g/dl; p = 0.31 and 39.7±3.9 vs. 40.4±4.2%; p = 0.53, respectively), and (3) the presence of clinical disease activity at time of sample procurement (yes vs. no: 13.7±2.1 vs. 13.7±1.3 g/dl; p = 0.98 and 39.7±4.7 vs. 40.0±3.7%; p = 0.78, respectively).

**Figure 1:**
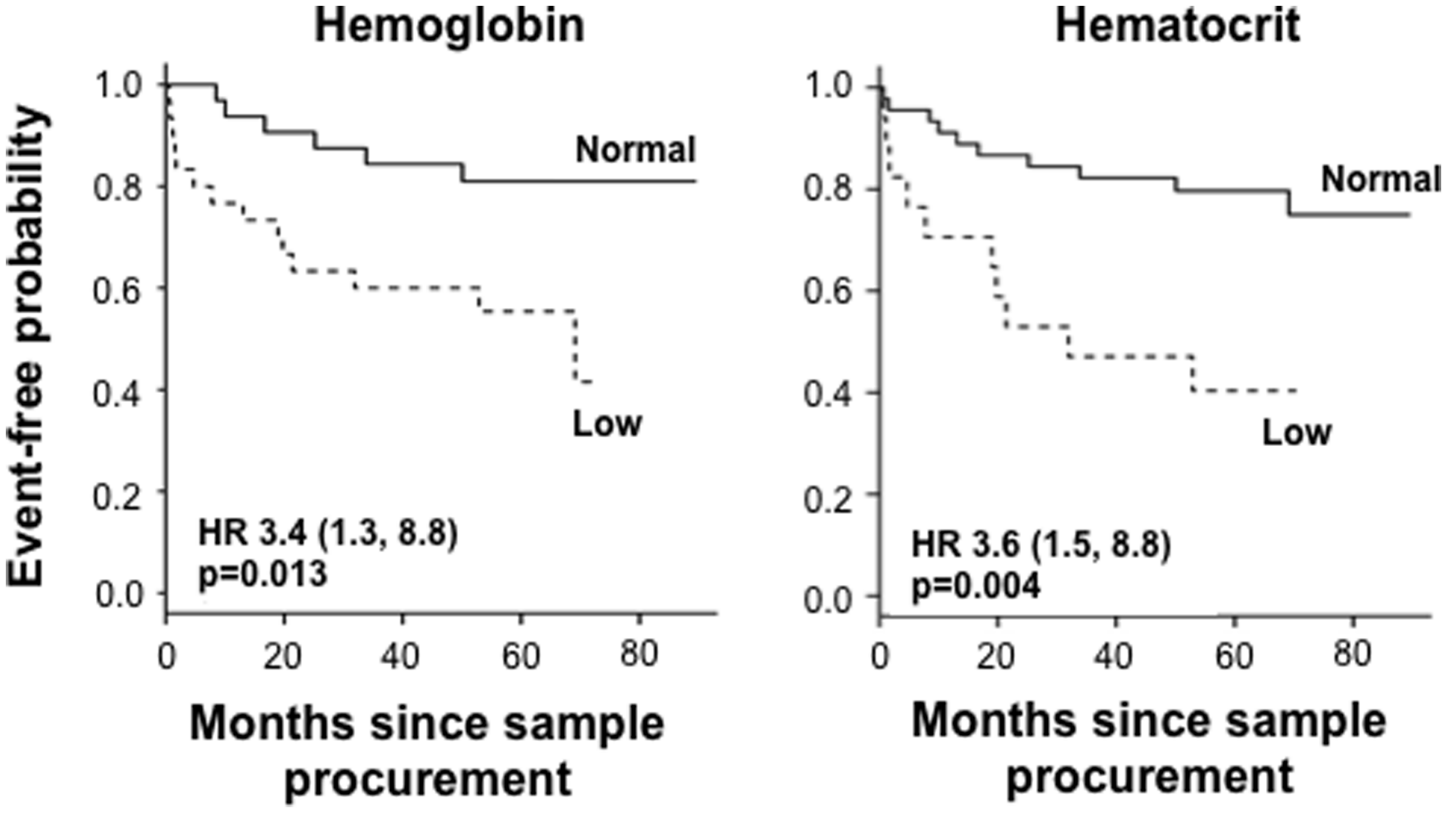
Hemoglobin (Hgb) and hematocrit (Hct) in the prediction of complicated Crohn's disease - Kaplan-Meier survival analysis for event-free probability. The broken line represents the population with low Hgb (<14.2 g/dl for males and <13.3 g/dl for females) or low Hct (<40% for males and <37% for females), respectively. The full line represent the population with normal Hgb or Hct. P-values and Hazard ratios (HR) as indicated in the figure.

The presence of low Hgb (Hazard ratio (HR) 3.4; 95% confidence interval (CI) 1.3, 8.8; p = 0.004) or low Hct (HR 3.6; 95% CI 1.5, 8.8; p = 0.013) levels at study entry (first sample procurement/baseline) predicted a faster progression to a first event, defined as fistula, stenosis or CD related surgery, in an unadjusted time to event analysis (**table 2** **;** **figure 1**). We then separately analyzed the occurrence of complications *or* CD related surgery during follow-up in the patient groups. In an unadjusted analysis a low Hgb or low Hct indicated a higher risk for early complications *or* CD related surgery.

**Table 2:** Unadjusted time-to-event analysis.

Factor	Complication and/or surgery (event)		Complication		Surgery	
	Hazard Ratio (95% CI)	p-value	Hazard Ratio (95% CI)	p-value	Hazard Ratio (95% CI)	p-value
Low Hct	3.6 (1.5, 8.8)	***0.004***	3.4 (1.3, 8.5)	***0.011***	4.7 (1.5, 14.8)	***0.008***
Low Hgb	3.4 (1.3, 8.8)	***0.013***	3.7 (1.3, 10.5)	***0.014***	4.1 (1.1, 14.9)	***0.032***

CI: confidence interval; p-values of <0.05 are bold and italic; Hct = Hemtocrit; Hgb = Hemoglobin.

Low Hgb: <14.2 g/dl for males and <13.3 g/dl for females.

Low Hct: <40% for males and <37% for females.

To further evaluate the predictive ability of the markers we used a Cox-proportional Hazard regression model taking into consideration as potential confounders: ileal disease location, clinical disease activity, disease duration, CRP positivity and age at diagnosis. Separate models were fit correcting for one potential confounder at a time due to the restricted patient number. Low Hgb levels or low Hct levels independently indicated a faster progression to an event, defined as the occurrence of complications or CD-related surgery (**table 3**), which was true for all tested potential confounders with hazard ratios between 3.0 and 3.9. Of note Hgb and Hct were not time-dependent covariates. The predictive ability in our cohort was independent of the time from sample procurement to event or in other words the link between a low Hgb or low Hct with the occurrence of events was present for early as well as late events after sample procurement. This was true for all examined time points (event in <16 months from sample procurement, >16 months, >24 months, >32 months and >48 months; data not shown). We additionally evaluated a possible link between need for iron therapy and complicated CD. Neither iron therapy at time of first sample procurement (HR 2.6; 95% CI 0.75, 8.8; p = 0.13), nor iron therapy at any time during follow-up (HR 1.7; 95% CI 0.69, 4.2; p = 0.25) were associated with complications and/or need for surgery.

**Table 3:** Adjusted time-to-event analysis.

Factor	Adjusted for Ileum Involvement		Adjusted for Active Disease		Adjusted for Disease Duration		Adjusted for CRP level			Adjusted for Age at Dx	
	Hazard Ratio (95% CI)	p-value	Hazard Ratio (95% CI)	p-value	Hazard Ratio (95% CI)	p-value	Hazard Ratio (95% CI)		p-value	Hazard Ratio (95% CI)	p-value
Low Hct	4.0 (1.6, 9.8)	***0.002***	3.1 (1.3, 7.6)	***0.013***	3.6 (1.5, 8.8)	***0.004***	3.0 (1.2, 7.4)		***0.016***	3.6 (1.5, 8.8)	***0.004***
Low Hgb	3.4 (1.3, 9.0)	***0.013***	3.9 (1.5, 10.4)	***0.006***	3.4 (1.3, 8.9)	***0.019***	3.0 (1.1, 7.8)		***0.028***	3.3 (1.3, 8.8)	***0.014***

CI: confidence interval; p-values of <0.05 are bold and italic; Hct = Hemtocrit; Hgb = Hemoglobin.

Low Hgb: <14.2 g/dl for males and <13.3 g/dl for females.

Low Hct: <40% for males and <37% for females.

As Hgb and Hct levels are subject to fluctuation over time we next analyzed serial measurements in our patients over time. 34 out of 62 patients had more than one sample taken during visits to our hospital throughout the disease course (2–7 samples per individual patient, median time between samples was 7 months (25^th^ percentile 3.2 months, 75^th^ percentile 13.6 months). The subgroups of patients with multiple visits compared to patients with only one visit were comparable in respect to age at study, age at diagnosis, the occurrence of complications, CD related surgery as well as time from sample procurement to first complication and CD related surgery. There was a significantly shorter disease duration, higher proportion of patients using immunosuppressants at the time of sample procurement and steroids at any time during follow-up as well as upper GI-tract location in the multiple visits group compared to the single visit group (**table S1**).

**Figure 2:**
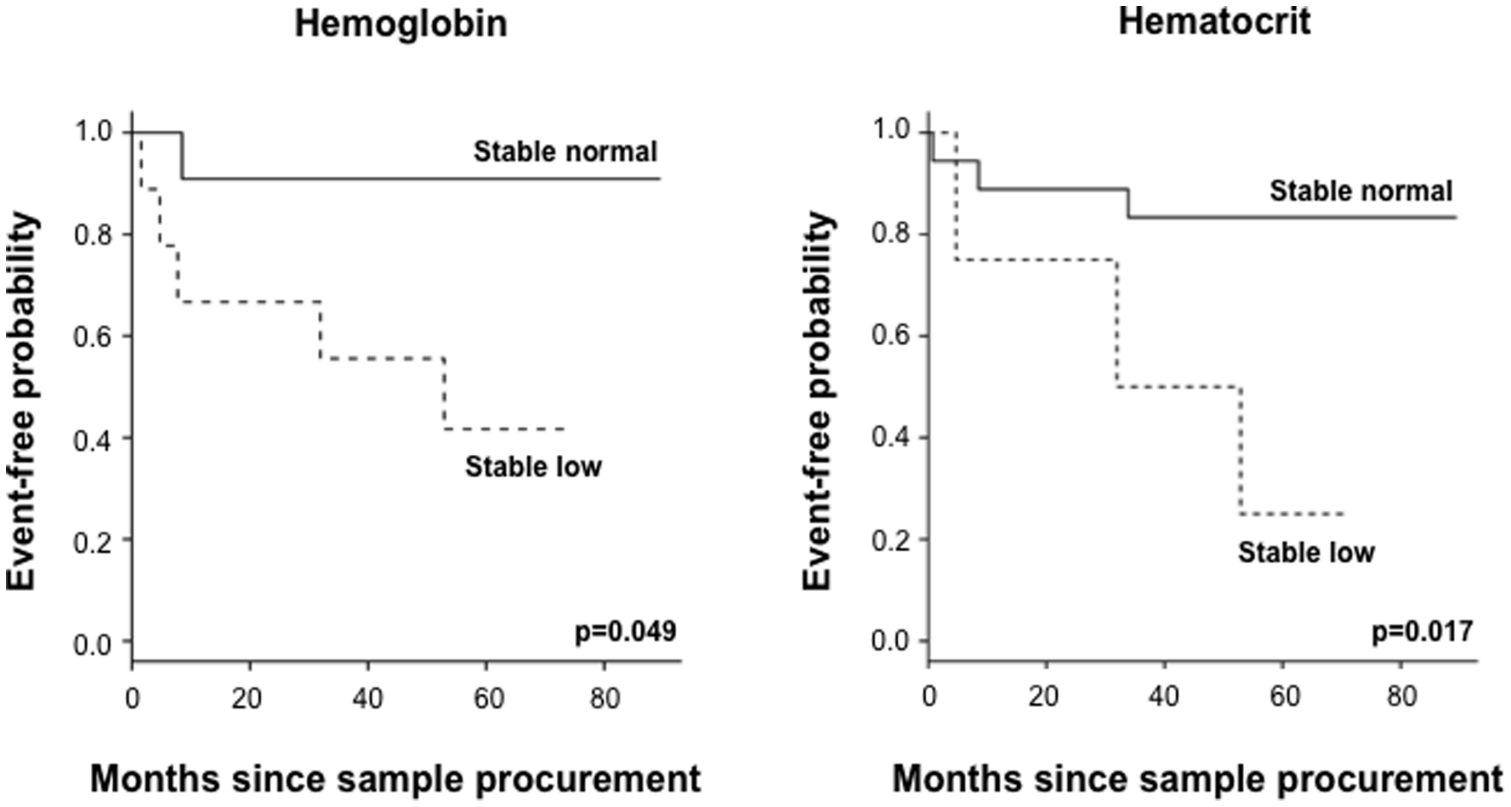
Serial measurements of hemoglobin (Hgb) during follow-up and association with complicated Crohn's disease - Kaplan-Meier survival analysis for event-free probability. The broken line represents the population with low Hgb (<14.2 g/dl for males and <13.3 g/dl for females) or low Hct (<40% for males and <37% for females), respectively. The full line represent the population with normal Hgb or Hct. P-value as indicated in the figure.

59% of CD patients had no change in their Hgb levels (normal versus low) and 35% had no change in their Hct levels on multiple visits during follow-up. We then compared the CD patients with a stable low Hgb or Hct with patients with a stable normal Hgb or Hct, respectively (**table 4** **,** **figure 2**). Patients with a stable low Hgb or Hct had a higher frequency of complications and events (complication and/or CD-related surgery) compared to CD subjects with stable normal Hgb or Hct values. In addition CD patients with a stable low Hgb had a shorter time to complication compared to stable normal Hgb.

**Table 4:** Hemoglobin & Hematocrit with Multiple Samples During Follow-up.

Factor	Stable Low Hct (n = 4)	Stable Normal Hct (n = 18)	p-value	Stable Low Hgb (N = 9)	Stable Normal Hgb (N = 11)	p-value
**Complication**	3 (75.0)	3 (16.7)	***0.046***	5 (55.6)	1 (9.1)	***0.049***
**Surgery**	2 (50.0)	2 (11.1)	0.14	3 (33.3)	1 (9.1)	**0.28**
**Complication and/or Surgery (Event)**	3 (75.0)	3 (16.7)	***0.046***	5 (55.6)	1 (9.1)	***0.049***

Hct = Hematocrit; Hgb = Hemoglobin.

p-values correspond to t-test for age and BMI, Wilcoxon rank sum test for disease duraiton and follow-up time and Fisher's Exact tests otherwise.

Clinical predictors for disease progression in complication naïve CD patients have been described, such as ileal disease location, disease duration, age at disease onset or positivity for ASCA IgG [9], [15]. We therefore tested, if the addition of Hct or Hgb to these factors can enhance the predictive capability (**table 5**). The AUC (Area under Receiver Operating Characteristics curve) for disease prediction of a low Hgb or low Hct was in the range of 0.66, which was higher compared to serology or clinical factors. When combining serology and clinical factors with Hct or Hgb the AUC increased to >0.7.

**Table 5:** Comparison of Hemoglobin and Hematocrit for the Prediction of Complicated Crohn's disease with ASCA IgG and Clinical Parameters.

ROC Model	AUC (95% CI)
Low Hgb	0.660 (0.532, 0.787)
Low Hct	0.667 (0.541, 0.793)
ASCA IgG	0.638 (0.520, 0.756)
Ileal involvement	0.557 (0.465, 0.649)
Age at disease onset	0.593 (0.430, 0.756)
Disease duration	0.563 (0.398, 0.728)
Low Hgb+ASCA IgG	0.724 (0.585, 0.862)
Low Hgb+Ileum involvement	0.686 (0.551, 0.821)
Low Hgb+Age at onset	0.696 (0.548, 0.845)
Low Hgb+Disease duration	0.667 (0.505, 0.828)
Low Hct+ASCA IgG	0.736 (0.604, 0.868)
Low Hct+Ileum involvement	0.695 (0.563, 0.828)
Low Hct+Age at onset	0.711 (0.566, 0.855)
Low Hct+Disease duration	0.675 (0.514, 0.836)

Hgb = Hemoglobin; Hct = Hematocrit; ASCA = Anti Saccharomyces cervisiae antibody.

AUC: Area under Receiver Operating Characteristics (ROC) curve; CI: confidence interval.

Low Hgb: <14.2 g/dl for males and <13.3 g/dl for females.

Low Hct: <40% for males and <37% for females.

For ASCA a cutoff of 35.1 IU was used.

## Discussion

We describe the use of Hgb and Hct for the prediction of complicated disease behavior or CD related surgery (defined as an event) in patients with CD. A low Hgb or Hct determined close to diagnosis independently indicated a higher hazard for the earlier occurrence of a complicated disease event. Hgb and Hct were not time dependent covariates. Patients with a stable low Hgb or Hct during serial follow-up visits had a higher frequency of events compared to patients with a stable normal Hgb or Hct, respectively. The addition of Hgb or Hct to commonly used clinical or serologic predictors can enhance the accuracy of disease stratification.

Anemia, indicated by low Hgb or Hct values, can be a trait of chronic inflammatory disorders, and occurs frequently in patients with CD [16]–[18]. Approximately 10% to 70% of subjects with CD develop anemia during their disease course [19]. The most common underlying cause for anemia in CD is multifactorial, with the major factors being iron deficiency anemia and anemia of chronic disease or both [20]. Studies have shown that anemia can be a potentially helpful tool for diagnosis of IBD [16]. Anemia appears to be related to chronic disease activity in CD [20] and is associated with an increased need for hospitalizations [19]. In addition markers related to erythrocytes, such as the red cell distribution width, have been linked to IBD activity [10]–[12]. Our cohort is in concordance with the literature with >60% of the patients having either iron deficiency anemia or anemia of chronic disease, with a minority being related to Vitamin B12 or folate deficiency or renal anemia.

Studies of this kind are of special importance as the course of CD is highly variable with patients changing from a pure inflammatory disease pattern to complicated disease behavior potentially at any time. Identifying markers to stratify patient groups at risk for developing certain disease phenotypes can make a tailored therapy possible. Several cross sectional, as well as prospective studies have shown a link between a profound serum immune response to microbial components and the occurrence of complicated disease courses [4], [5], [7]–[9]. Limited information is available on routine blood tests such as Hgb and Hct and outside of this investigation there is no study available evaluating Hgb for this matter. Our study shows that blood tests routinely used for CD patients in a clinical setting can aid as predictors of a more severe disease course, defined as the earlier occurrence of fistulae, stenoses or CD related surgery. Importantly, this was true for early events after sample procurement as well as later events, such as those occurring more than two years after sample procurement. The addition of Hgb and Hct to clinical or serologic factors increased their predictive accuracy. Interestingly this effect was independent of disease acitivity, serum CRP, ileal involvement or disease duration. Our data therefore indicates that a low Hgb or Hct value early in the disease course could be an additional tool on top of a thorough clinical workup that can help identifying CD patients at risk for complications.

Determination of Hgb and Hct is easy and rapid to perform, cheap and well reproducible and is part of standard of care laboratories in IBD patients. The lack of specificity of anemia for CD can be neglected once diagnosis is made on clinical grounds. Hct and Hgb fluctuate over time. However, about half of the CD patients in whom multiple samples throughout the disease course were available remained either normal or low for Hgb and Hct. This could indicate that despite fluctuations certain patients have a predisposition to lower Hgb or Hct values indicating more severe disease or normal Hgb or Hct values indicating less severe disease [20]. This can be explained by anemia of chronic disease or increased blood loss due to intestinal ulcerations. Patients with multiple visits had a significantly higher rate of upper GI involvement, a higher intake of immunosuppressants, but the same amount of complications and surgeries compared to patients with only one visit. One possibility for multiple visits despite the same amount of complications is the need to monitor immunosuppressive medications or the increased rate of upper-GI involvement.

Interestingly, patients with active CD have significantly impaired intestinal iron absorption [21]. Iron absorption was negatively correlated with serum CRP and IL-6 levels in a pediatric CD population. It has been hypothesized that IL-6, released by the human intestine, induces hepcidin in human hepatocytes that in return inhibits intestinal iron absorption [21]. Interestingly, in our cohort neither CRP nor clinically active disease at time of sample procurement were linked to low Hgb or Hct levels. This could be explained by a possibly delayed effect of active disease on reduction in Hgb/Hct or by the limitations of CRP or clinical disease activity in depicting actual intestinal mucosal damage. The application of iron therapy was not linked to complications or need for surgery. The missing association could be due to the low patient number of this study in combination with the multifactorial etiology of the anemia in this population.

However, certain limitations apply: Our clinic is a regional referral center for IBD and therefore our patient population represents more severe disease courses with a relatively high amount of complicated disease behavior, CD related surgery and ileal disease location. This could introduce a referral bias. The majority of the IBD patients were evaluated by four experienced IBD physicians using pre-set criteria. We cannot exclude that few patients included in the study were evaluated by additional GI certified providers. However criteria used were identical for all included subjects. This study is a cohort study and not a controlled prospectively designed study. We are aware of the potential presence of a subclinical complication at the time of sample procurement, which might explain the early occurrence of complication or surgery after sample procurement. 50% of the samples were taken more than one year after diagnosis, which could represent a different population compared to studies using samples strictly taken at time of diagnosis only. The sample size is moderate. A larger cohort would enable a more detailed analysis in respect to type of complications, such as separating perianal fistulizing and internal penetrating disease courses or separating the type of anemia. None of the patients had fecal calprotectin measured at time of serum sample procurement or during the disease course. We did not have the exact duration of treatment that was administered during the follow-up period and therefore did not perform a more detailed analysis of the impact of therapies on Hgb and Hct levels. Nevertheless, we believe that our data support the concept of a predictive ability of the blood tests Hgb and Hct for complicated CD behavior and CD related surgery.

## Conclusion

Decreased levels of Hgb or Hct could serve as additional indicators for a more rapid progression towards a complicated CD behavior or CD related surgery. This study needs to be independently validated in a larger prospective study.

## Supporting Information

Table S1 **Comparison of subgroups with one visit or multiple visits.**
(DOCX)
