# Supplementary material for: Hemoglobin and Hematocrit Levels in the Prediction of Complicated Crohn's Disease Behavior – A Cohort Study
Source: PLoS One. 2014 Aug 12;9(8):e104706. doi: 10.1371/journal.pone.0104706 (PMC4130535; doi:10.1371/journal.pone.0104706)
Supplement: Table S1 — Comparison of subgroups with one visit or multiple visits. (DOCX) [file pone.0104706.s001.docx]

| **Table S1: Comparison of subgroups with one visit or multiple visits** | | |  |  |
| --- | --- | --- | --- | --- |
| **Factor** | **Multiple Visits (N=34)** | **One Visit (N=28)** | **p-value** |  |
| Female, n (%) | 17 (50.0) | 16 (57.1) | 0.57 |  |
| Mean age at study (SD) | 31.6 ± 10.3 | 33.7 ± 13.6 | 0.50 |  |
| Mean BMI (kg/m2) (SD) | 22.9 ± 3.6 | 23.1 ± 3.8 | 0.79 |  |
| Mean age at diagnosis (SD) | 29.4 ± 9.8 | 29.7 ± 12.4 | 0.91 |  |
| Median disease duration (months) (P25, P75) | 9.1 [0.9, 36.6] | 25.8 [4.2, 73.5] | ***0.046*** |  |
| Location (non-exclusive) |  |  |  |  |
| Upper GI-tract (%) | 10 (29.4) | 2 (7.1) | ***0.027*** |  |
| Jejunum, prox. Ileum (%) | 1 (3.0) | 0 (0.0) | 0.99 |  |
| Ileocoecal (%) | 5 (14.7) | 8 (28.6) | 0.18 |  |
| Colon w/o Cecum (%) | 5 (14.7) | 6 (21.4) | 0.49 |  |
| Ileum and Colon (%) | 24 (70.6) | 14 (50) | 0.098 |  |
| Small Intestine (%) | 5 (14.7) | 6 (21.4) | 0.49 |  |
| Rectum (%) | 6 (18.2) | 4 (14.3) | 0.68F |  |
| Ileum involvement | 29 (85.3) | 22 (78.6) | 0.49 |  |
| Vienna Classification |  |  | 0.72F |  |
| Inflammatory | 23 (67.7) | 21 (75.0) |  |  |
| Stricturizing | 4 (11.8) | 4 (14.3) |  |  |
| Fistulizing | 7 (20.6) | 3 (10.7) |  |  |
| Montreal classification |  |  | 0.52F |  |
| B1 | 23 (67.7) | 21 (75.0) |  |  |
| B1p | 5 (14.7) | 1 (3.6) |  |  |
| B2 | 4 (11.8) | 4 (14.3) |  |  |
| B3 | 2 (5.9) | 2 (7.1) |  |  |
| Use of Immunosuppresants at time of sample procurement | 28 (82.4) | 15 (53.6) | ***0.014*** |  |
| Use of Steroids at any time during follow-up | 25 (73.5) | 10 (35.7) | ***0.003*** |  |
| Complication | 11 (32.4) | 7 (25.0) | 0.53**^c^** |  |
| Surgery | 8 (23.5) | 5 (17.9) | 0.59**^c^** |  |
| Complication and/or Surgery | 12 (35.3) | 8 (28.6) | 0.57**^c^** |  |
| Median time from sample procurement to 1st event or last FU (P25, P75) | 61.0 [19.0, 72.7] | 53.2 [41.2, 61.7] | 0.5 |  |
| BMI, body mass index | | | | |
| P25, P75: 25th and 75th percentiles; SD: standard deviation; FU: follow-up | | | | |
| Values presented as Mean ± SD, Median [P25, P75] or N (column %). | | | | |
| F=Fisher's Exact test. | | | | |
